# Older patients’ and their family caregivers’ perceptions of food, meals and nutritional care in the transition between hospital and home care: a qualitative study

**DOI:** 10.1186/s40795-020-00335-w

**Published:** 2020-03-18

**Authors:** Christine Hillestad Hestevik, Marianne Molin, Jonas Debesay, Astrid Bergland, Asta Bye

**Affiliations:** 1 Department of Physiotherapy, Faculty of Health Sciences, OsloMet - Oslo Metropolitan University, Oslo, Norway; 2 Department of Nursing and Health Promotion, Faculty of Health Sciences, OsloMet - Oslo Metropolitan University, Oslo, Norway; 3 Bjorknes University College, Lovisenberggata 13, 0456 Oslo, Norway; 4 European Palliative Care Research Centre (PRC), Department of Oncology, Oslo University Hospital and Institute of Clinical Medicine, University of Oslo, Oslo, Norway

**Keywords:** Older persons, Family caregivers, Nutrition, Hospital, Home care, Person-centred care, Transitional care

## Abstract

**Background:**

Older people have varying degrees of unmet nutritional needs following discharge from hospital. Inadequate involvement of the older person and his or her family caregivers in care and care planning, and inadequate support of self-management in the discharge process and follow-up care at home, negatively affects the quality of care. Research on older patients’ and their family caregivers’ experiences with nutritional care in hospital and home care and in the transition between these settings is limited. Thus, the aim of this study was to explore older patients’ and their family caregivers’ perceptions regarding the food, meals and nutritional care provided in the transition between hospital and home care services, focusing on the first 30 days at home. The overall aim of this study is to produce knowledge that can inform policy and clinical practice about how to optimise the care provided to older persons that are malnourished or at risk of malnutrition.

**Methods:**

Using a qualitative interpretive descriptive design, we carried out face-to-face semi-structured interviews with 15 older patients, with documented risk of malnutrition or malnourishment (Mini Nutritional Assessment [MNA]), two and five weeks after hospital discharge. In addition, we interviewed nine family caregivers once during this five week period. The questions focused on perceptions of food, meals and nutritional care in hospital and home care and in the transition between these settings. We analysed the data thematically.

**Results:**

Four overarching themes emerged from the material: 1) the need for a comprehensive approach to nutritional care, 2) non-individualised nutritional care at home, 3) lack of mutual comprehension and shared decision making and 4) the role of family caregivers.

**Conclusion:**

The organisation of nutritional care and food provision to older people, depending on care, lack consideration for the individual’s values, needs and preferences. Older patients’ and their family caregivers’ needs and preferences should guide how nutritional care is provided.

## Background

Adequate nutrition is a cornerstone of good health, but food and meals are also strongly connected to identity, social and cultural life; they are important for every individual’s quality of life. Food may also add a sense of meaning, security and structure to a person’s day and provide feelings of dignity, independence and control [1–3]. However, many older patients find that eating becomes a challenge. Physiological, psychological and social changes due to aging may lead to loss of appetite, eating difficulties and diminishing food intake [4, 5]. Persistent insufficient intake of food and nutrients develops into malnutrition, which is associated with a number of adverse consequences such as impaired physical and psychological function, reduced quality of life, prolonged hospital stays, increased readmission rates and increased healthcare costs [3, 6]. Studies report that up to 60% of older medical patients are malnourished or at risk of malnutrition when they are admitted to hospitals [7, 8]; their nutritional status often declines during their stay [9]. Studies also report malnutrition and risk of malnutrition among 14.6–56% of older people receiving home care [7, 10].

Older people are particularly vulnerable to malnutrition due to several factors such as age-related pre-disposition to acute diseases, chronic diseases and comorbidities, side effects of medications, advanced age and hospitalisation [4, 5]. The transition from the hospital to the community also seems to be critical in the development of malnutrition in the home dwelling due to inadequate nutritional care [5, 11]. Poor communication of nutritional information and failure to coordinate care when patients are transferred between different levels of care are common [3, 12]. Inadequate food availability and quality [13], insufficient knowledge about nutritional needs and lack of attention to nutritional problems among healthcare professionals have also been described [14, 15]. Another common problem is poor communication with and lack of involvement of the older patient in his or her own health and nutritional care planning [16]. The same applies to family caregivers’ involvement, which may be crucial for shorter hospital stays, preventing hospital readmission and improved satisfaction with the healthcare experience [17–19].

Healthcare in Norway is predominantly state-funded and available to all residents. Hospitals are mainly responsible for acute and specialised healthcare services, while the municipalities provide healthcare that is suitable for the early identification and follow-up of somatic and mental health [20]. Institutional closure and rationalisation of the hospital/nursing home sectors has caused an increased number of elderly patients being discharged to their homes with home care services instead of institutional care. The municipality administers these services to people who require help for either a short or long period as a result of illness, impaired health, old age or other factors. Municipal healthcare services are obliged to provide individualised care, including information, advice and counselling, to prevent disease, injury and social problems. The care includes practical help with hygiene, cooking and cleaning and home nursing (medication, wound treatment and nutrition) [21].

When organising nutritional care, it is important to consider older patients’ and their family caregivers’ perspectives [22, 23]. There seems, however, to be limited research on older patients’ and their family caregivers’ experiences with nutritional care in hospital and home care and in the transition between these settings. Thus, this study aims to explore these groups’ perceptions regarding the food, meals and nutritional care provided in the transition between hospital and home care services, focusing on the first 30 days at home.

## Methods

This study was part of a larger project focused on cross-sectoral care transitions for older patients from specialized hospital care to municipal health and care services, including the first 30 days post discharge. The first period following hospital discharge is a vulnerable time for patients [24], and their journey between healthcare sectors may provide important information that can be used to improve the quality of transitional care.

### Design

To explore the experiences of the older patients and their family caregivers, we used an interpretive descriptive approach described by Sally Thorne et al [25]. This is an inductive analytic approach, designed to create ways of understanding clinical phenomena, which yield knowledge relevant for the clinical context of applied health disciplines. Between May 2017 and June 2018, we carried out individual semi-structured interviews with older patients and their family caregivers to capture their experiences of food, meals and nutritional care provided in the transition between hospital and home care services.

### Data collection

We recruited a purposive sample [26] of patients aged 65 and above from an acute geriatric care ward in a large hospital in Norway. In order to get participants with experiences of the phenomena being studied, eligible patients had to be in need of nutritional care. Therefore, we only asked patients classified as malnourished or at risk of malnutrition according to the Mini Nutritional Assessment (MNA) [27] to participate. They also had to be discharged to their homes with home care nursing services and be able to give informed consent. The patients need for nutritional care at home was evaluated by the municipality after discharge.

The nursing staff in hospital informed the first author when eligible patients were admitted. She approached the patients, informed them about the study and asked if they were willing to participate.

We conducted the interviews twice, at two and five weeks after transition from the hospital in order to capture their recent experiences from the actual transition and to get information about their experiences from the whole 30-day period. In qualitative studies, 15 ± 10 participants is considered as a sufficient sample size to get information about the research phenomena and to yield a manageable amount of data [28]; we aimed to recruit 15–20 patients or continue recruitment until we reached data saturation [26]. We also interviewed family caregivers once at 2–5 weeks after discharge. Originally we wanted to interview patients and family caregivers separately, but when there was a wish for them to be interviewed together this was allowed.

### The characteristics of participants

We invited 23 hospital patients to participate in the two interviews; of these, one patient declined. We phoned the patients a few days before each interview to arrange a time for the interviews. At this time, four of the patients withdrew from the study, which resulted in 18 included patients. Additionally, we excluded three patients because they had not been transferred to their homes, but to a municipal service such as intermediate care or a nursing home. This resulted in 15 included patients. One patient died before the second interview, one we did not contact for a second interview because the interview situation seemed to cause anxiety and two could not be reached, resulting in 11 participants whom we interviewed twice. We conducted all but two interviews in the patients’ homes (one by phone and one in a cafeteria close to home in line with the patient’s wishes). Twelve of the included patients had family caregivers who helped them with nutritional care on a regular basis; we asked them to participate. One declined and two withdrew from the interview due to health problems, resulting in nine included caregivers. We interviewed the patients and their family caregivers separately except for two instances, in which we interviewed both the patient and caregiver together based on their wishes. After interviews with 15 patients and nine family caregivers no new insights relevant to the study topic seemed to be revealed, and data was considered to have reached saturation. Altogether, the first author performed 33 semi-structured interviews with 15 older patients and 9 of their family members. The interviews lasted 25–90 min.

The participants’ characteristics are presented in Table 1. The patients were comprised of 10 women and 5 men aged 74–97 years (mean age: 85 years) with several diseases or other health conditions. The family caregivers (6 women and 3 men) were the patients’ spouses, children and grandchildren and were aged 52–86 years (mean age: 65 years). All of the participants lived in the same urban area in Norway.

**Table 1:** Characteristics of the participants

Patient (P)	Age range	Gender	MNA score^a^	Hospital days	Reasons for hospital admission	Living-situation	No. of inter-views	Family caregiver (FCG) (age)
1	91–95	F	16	5	Worsened heart failure, back pain	Alone	2	Grand-daughter (52)
2	81–85	F	20,5	6	Malfunction and impaired general health condition	Alone	1	
3	71–75	F	14	6	Neurological problems, hypokalemia, chronic obstructive pulmonary disease (COPD), portal hypertension, liver cirrhosis	Alone-sheltered housing^b^	2	
4	91–95	F	17,5	7	Frailty, infection, cancer	With spouse	1	
5	76–80	M	14	6	Urinary tract infection, Parkinson’s, kidney failure, dysphagia	With spouse	2	Wife (83)
6	91–95	F	22,5	4	Broken arm, fall tendency, mild cognitive failure	Alone	1	Daughter (54)
7	81–85	M	9,5	7	Pain in the jaw, severe weight loss	With spouse	2	Wife (80)
8	86–90	F	17	4	Pyelitis, weight loss, lack of appetite, nausea	Alone	2	Daughter (58)
9	76–80	M	18,5	10	Sepsis, pneumonia, metastatic prostate cancer, severe weight loss	With spouse	2	
10	81–85	F	16	3	Loss of consciousness, atrial fibrillation, back pain	With spouse	2	Husband (86)
11	86–90	M	17	3	Chest pain, lack of appetite, difficult home situation	Alone	2	
12	96–100	M	14	5	Fall/ailment, heart failure, urinary tract infection, dysphagia	Alone	1	
13	81–85	F	11	5	Pneumonia, COPD, severe malnutrition	Alone	2	Daughter (61)
14	81–85	F	16.5	5	Fall, loss of consciousness, lack of appetite, weight loss	Alone in sheltered housing^b^	2	Son (55)
15	76–80	F	20	5	Pulmonary embolism/aortic plastanosis, diabetes type 2 (insulin regulated)	Alone	2	Son (56)

^a^Total MNA assessment (part 1 + 2): 17.0 to 23.5: At risk of malnutrition; less than 17 points: malnourished. Measured in hospital

^b^Housing with available care services including dinner service in a cafeteria

### Interview guide

We used interview guides to direct the interviews toward the participants’ experiences of food meals and nutritional care and to allow for unexpected themes and viewpoints to emerge. The questions were open-ended, and we asked probing questions to explore or clarify aspects that arose in the interviews. Examples of questions from the interview guide are presented in Table 2.

**Table 2:** Examples of questions in the interview guides

**The older patients**	**Interview two weeks after discharge:** How would you describe your nutritional situation? What are your needs when it comes to food and meals after hospital discharge? Can you please describe the nutritional care you received in hospital? How do you think your needs and preferences were considered in this care? Can you describe the nutritional care you have received at home since you came home from hospital? How do you think your needs and preferences are considered in the nutritional care at home? Can you describe your collaboration with the home care services? How do family caregivers contribute to your nutritional care? Can you describe how you collaborate with your family caregivers in nutritional care? How do you think the nutritional care could be improved? **Interview 5 weeks after discharge:** How would you describe your nutritional situation now? Have there been any changes to the nutritional care you receive? If yes, describe them. How do you feel your needs and preferences are met now? How could your situation with food, meals and nutritional care be improved?
**Family caregivers**	**Interview 2–5 weeks after discharge of family member** Can you please describe your family member’s needs for nutritional care? How do you perceive these needs are met? How do you contribute to his or her nutritional care? How would you describe your communication and collaboration with the care services? How do you collaborate with your family member in nutritional care? How do you think the nutritional care to your family member could be improved?

### Data analysis

We audiotaped and transcribed the interviews, reviewed the transcriptions for accuracy and then anonymised them. We used NVivo for data management, coding and data analysis. We used a thematic coding technique based on Braun and Clarke’s [29] framework; this is a method for identifying, analysing and reporting patterns within qualitative data. This method includes six phases: (1) familiarisation with the data, (2) creating initial codes (3) searching for themes across the data, (4) reviewing the themes, (5) defining and naming the themes; and finally (6) writing the report. Examples of the coding strategy is presented in Table 3.

**Table 3:** Examples of coding strategy

Quotation	Initial code	Sub-theme	Overarching theme
‘It is dreadfully boring to eat alone. It really is!’ (P3)	Lonesome meals	Psychosocial needs related to food and meals	The need for a comprehensive approach to nutritional care
‘P: Frequently I don’t eat on Saturdays because I’m not fond of porridge. I: Is there no other alternatives? P: No, (on Saturdays) we only get porridge’. (P14)	Being served food she dislikes	Lack of consideration for individual needs	Non-individualised nutritional care at home
‘I’ve been sitting here waiting for them to turn up three evenings in a row, and now I am fed up’. (P2)	Tired of having to wait for home care to turn up	Respect my time!	
‘A: She was standing there with her back towards me preparing the food. I asked her something and she didn’t reply. I am used to people responding, so I repeated the question. Then, she said, “I heard you the first time”. I then replied, would you please just give me a response’. (P13)	Not being heard	What about my opinion?	Lack of mutual comprehension and shared decision making
‘I: Can you remember the conversation with the nutritionists? P: No I can’t remember much, I wasn’t able to pay attention because I wasn’t feeling well’. (P9)	Getting a consultation when feeling too poor to pay attention.	It was so complicated I just gave up!	
‘I called the home care services to tell them to admit my husband to a rehabilitation institution, because I was exhausted and couldn’t take it anymore. Or else, they would have to admit me as well’. (FC7)	Being exhausted from caring for her husband	Lack of support to the caregivers	The role of family caregivers

To maximise trustworthiness and limit threats to validity, we employed the criteria for ‘trustworthiness’ outlined by Lincoln and Guba [30]. We met the criterion for credibility through open-ended questioning, prolonged engagement with the data and by providing a detailed description of the methods. We fulfilled the criterion for transferability through presenting detailed and in-depth descriptive data and by quoting the participants. To meet the criterion for dependability, each transcription was independently read, checked and coded by three of the authors; and final interpretations were reached via agreement among all five authors. We employed the criterion of confirmability by providing rich quotes from the participants depicting each theme. We also follow the consolidated criteria for reporting qualitative studies (COREQ) [31].

### Ethical considerations

The Norwegian Regional Ethics Committee approved this study (2016/2215 REK B). All of the patients were deemed able to give informed consent by healthcare personnel in hospital. The first author approached the participants in hospital and introduced herself as a PhD student with a nutritional education. She presented the participants with verbal and written information outlining the purpose of the study and told them that participation was voluntary. We obtained written informed consent from all of the participants and guaranteed their confidentiality. We also informed them of their right to withdraw from the study at any stage and assured them that this would not affect their current or future access to services.

## Results

We present our findings through four overarching and interrelated themes and sub-themes that emerged from the interviews (see Table 4).

**Table 4:** Themes and sub-themes emerging from the interviews

The need for a comprehensive approach to nutritional care	• Food and meals in the context of aging and disease • Psychosocial needs related to food and meals
Non-individualised nutritional care at home	• Lack of consideration for individual needs • Cooking is pressing a button on the microwave • Respect my time! • Striving for independence
Lack of mutual comprehension and shared decision making	• It was so complicated I just gave up! • What about my opinion?
The role of family caregivers	• Dependent on family caregivers support to cover needs • Lack of support to the family caregivers

### The need for a comprehensive approach to nutritional care

#### Food and meals in the context of aging and disease

Most of the patients found that their nutritional status was deteriorating. Their family caregivers agreed: ‘Her nutritional status is much poorer than it used to be. I think it’s due to her old age. She has lost a lot of weight the last 3 years; she eats less and is less active’ (FCG8). The patients described struggling with on-going health problems: ‘My health is still very poor because I have cancer in my whole body’ (P9). They also experienced new health problems: ‘Since I came home from the hospital, my mouth and tongue have suddenly become very sore. It really hurts and I can’t taste the food. I don’t understand what causes it’ (P5). All of the patients described challenges related to food and meals. Most of the patients described a lack of appetite, and many said they never felt hungry: ‘I eat and it doesn’t really taste bad, but. .. A lot of strange things have happened to me. It is just like I don’t feel much like eating anymore. I don’t know why. .. I probably don’t want to become fat again’ (P8). Some patients experienced reduced appetite due to nausea, and some had difficulties chewing or swallowing food due to tooth problems, dysphagia and sore mouth and tongue. Patients also described that food no longer tasted like it used to: ‘The food doesn’t taste [like] much. I have been wondering about that. .. I make ham with mayonnaise and spices, but it just doesn’t taste [like] anything. It may be due to the medications’ (P3). In general, the patients took many medications. Some of these had to be taken before food, some with food and some after food. Having to plan meals according to medications seemed to negatively influence their nutritional situations. They also described not being able to cook anymore because of functional limitations. Several patients made statements along the lines of ‘I only eat because I have to’. The patients understood that food was a necessity for their well-being, but the challenges described above made it difficult for them to ensure sufficient food intake: ‘I feel full so quickly, and I can’t force myself to eat, that can’t be right, that’s ridiculous’ (P8). One patient described becoming very ill because he could not eat enough: ‘I was very sick and felt so dizzy that I almost didn’t know where I was. This happened because I ate too little’ (P9).

Most of the patients had recently lost weight; this was a fact they were aware of, but for most, they were not worried. The weight loss only seemed to become problematic when it became visible to others or was related to practical problems such as not having any clothes to wear: ‘I don’t feel that I am undernourished. The only problem I can think of is that I don’t have anything to wear, because all my clothes are too big’ (P14).

#### Psychosocial needs related to food and meals

Many of the patients that lived alone experienced loneliness, and this seemed to affect their appetite and meal experience. Several patients described eating alone as depressing: ‘I am very fond of food, and when I eat homemade bread with butter and honey, I feel that my mood gets better. But I can be quite depressed at times. .. I think it is pretty lonely being here all alone’ (P6). Some also talked about their inability to participate in social activities, such as going to a cafeteria with friends or to dinners, because of restricted mobility*.* They talked about eating more when they ate together with someone; this was also the family caregivers’ impression. One patient was interested in eating at a senior centre but was put off by the idea because she had been told there would be many people with dementia there. Some of the patients occasionally ate at a local nursing home for variety from the meals at home and to socialise, but this was not necessarily a good experience: ‘A lot of the people there already know each other, and they sit together and talk. When I come from outside I don’t know anyone and end up sitting alone. That feeling of loneliness hurts. I eat my food fast and then I leave’ (P3).

### Non-individualised nutritional care at home

#### Lack of consideration for individual needs

Most of the patients were very pleased with the care they received in hospital. The healthcare personnel were focused on their nutritional needs and the nurses tried their best to provide them with food to their liking. Some of the patients had a consultation with a nutritionist during their stay and were prescribed nutritional drinks and instructions on how to enrich their meals. However, they did not seem to follow these instructions at home after discharge. The care at home seemed to lack respect or consideration for each patient’s unique needs and preferences concerning food and meals. One patient said, ‘No one has been here and told me that you need more of this and that and that we need to make your food richer in calories. It is more like, “What do you want?” And then that’s what you get’ (P13). None of the patients could remember home care services asking them about their nutritional problems, the reasons behind the problems or discussing possible solutions. They believed that many of the staff lacked competence about nutrition and knowledge about their nutritional needs and preferences. The home care staff were often students studying other fields than healthcare who were working part time in home care while studying. The patients said that qualified nurses seldom visited them: ‘The nurses seldom stop by. They are supposed to be here twice a week, but that doesn’t happen very often’ (P13). In general, the patients experienced few changes in their care between the first and the second interview.

#### Cooking is pressing a button on the microwave

The food they were served by home care usually consisted of slices of bread for breakfast and lunch and ready-made meals heated up in the microwave for dinner. Some of the patients thought this food was ok, but several of the patients disliked these meals: ‘I can actually make sandwiches on my own. I can’t stand the sandwiches they prepare for breakfast and put in the fridge the night before. The next morning, they are ice cold and I think they are horrible’ (P14). Especially, the food prepared for dinner was problematic for some. All the patients living alone described eating ready-made meals for dinner regularly, and they described cooking as ‘pressing a button on the microwave’. They felt that because these meals had to be made within the sparse time the home care services had for each of them, their diets were limited. One patient said, ‘I had to get a microwave oven, because that was the first thing they (homecare services) asked for when they started coming here. I don’t like such food. I want to make homemade food. I don’t know what kinds of food I am supposed to buy. I can’t eat only ready-made meals. First of all, I can’t stand the taste of these ready meals, and secondly, I find them too expensive’ (P1). The family caregivers explained that they bought these microwave meals for practical reasons even though they generally knew that the patients were not fond of them. Several patients described being served tasteless meals and food they disliked, which resulted in a reduced food intake: ‘We get some fish cakes without taste with grated carrot without any dressing, with potato and maybe a spoonful of butter. You don’t enjoy that. It doesn’t taste anything. Then I just eat a little and leave the rest’ (P3).

Many of the patients described a longing for tasteful meals; this was often traditional foods or food normally served during holidays. The patients also described pleasurable meal experiences: ‘Patient: You know, I get so happy when the food finally tastes good and we get a tasty sauce or something. Aah. .. today we had herring with onions, red beets and sour cream and I had two filets and two potatoes. Today the food was lovely and it made me happy. Interviewer: I’m glad to hear that you had a good meal. Patient: Yeah, that is so important for my wellbeing and everything’ (P3). Tasteful meals, meals made by family caregivers and eating in social settings seemed to stimulate the patients’ appetite and inspire them to eat more: ‘The other day I really ate too much for dinner. My daughter served fried sausages with red cabbage and potatoes and it was just so good!’ (P13).

#### Respect my time!

The patients reported that care was organised in a way that seemed to lack respect for their daily routines. Home care staff often did not turn up when they were supposed to, and patients described not being able to go out because they had to wait for staff, which restricted their freedom: ‘On Sundays, they phone me at quarter to twelve and say, “Hi, we are a bit delayed today”. They come to make me breakfast at quarter past twelve! It is a bit like that. It is often because of shortage of staff’ (P13). The patients also told stories of home care services not turning up at all. Additionally, when they tried to phone them, they could not reach anyone: ‘It is impossible to call them, and no one answers the phone during the day. No one. You can’t get hold of anyone’ (P13). One patient who was almost blind and unable to prepare food experienced this several times. Fortunately, she could call her daughter who lived close by for help. When home care staff did not show up or did not turn up at the right time, the patients were not in charge of their life situations*.*

Sometimes, the home care visits were scheduled too early or too late and were not in accordance with the patients’ preferences. When being dependent on help for meals, bad timing of the meals could result in patients not eating for many hours or eating less: ‘I don’t eat much besides dinner. I: At what time is that? P: At one o’clock, that is a ridiculous time. .. I set my alarm clock at 10–11, I then get up and sort out my medication and I’m not hungry, and then I just wait for dinner. If dinner had been at three o’clock instead, I would have made some breakfast’ (P3). The patients reported that the care services were always in a rush, not having time to care for them or talk to them: ‘For many people living alone, the only human conversation they have during a day is with the people from home care services. I think it is important that they take the time to talk to these people, not just rush in, make food and goodbye’ (P13).

#### Striving for independence

Most of the patients were striving to maintain independence and control, and they wanted to manage their food and meals themselves; however, they needed help. For some, becoming dependent on help from others was an inner struggle: ‘When you are used to managing everything yourself and then you suddenly become dependent on help to prepare a sandwich. Can you imagine? It is a bit depressing just thinking about it’ (P13). One patient valued her independence so much that she kept her problems with food and meals hidden from her family for a long time and rejected care services’ help. One of her family caregivers said, ‘She never told us about her problems. They reduced the home care from every two weeks to every third week. The weaker she got the less help she received. But then they told us in one of the meetings that she has been offered help several times, but she has not been interested. She prefers to manage on her own’ (FC14).

The patients emphasised the importance of having a voice in the matter of food and meals – ‘I want to have a say in the matter too’ (P15) – and the importance of being able to eat the food they wanted whenever they wanted. The patients who needed assistance preparing meals did not have this freedom; they had to eat what the care services could manage to prepare within their short timeframe.

Although the patients expressed a desire to be independent, they were not offered any self-management support from the care services. They were dependent on additional help from family caregivers, neighbours and friends to manage their daily lives, to understand information and to communicate with healthcare services, indicating a paternalistic approach to care. These disempowering experiences with home care resulted in dissatisfaction with care.

### Lack of mutual comprehension and shared decision making

#### It was so complicated, I just gave up

The patients described a lack of mutual comprehension of information about their health, treatment and care. They told stories about not understanding or remembering information they received in hospital. In fact, most of the patients who received a consultation with a nutritionist in hospital could not remember the content of this conversation: ‘Two nutritionists came to talk to me when I was in hospital. Interviewer: what did they say? Patient: I don’t know, it was so complicated, I just gave up’ (P9). The reasons they gave for this were that they were too ill, there were too many different people involved in their care and that they experienced medical terminology and information overload. Many of the patients were not able to understand the written information they received in hospital: ‘I received a lot of written information, but I don’t understand half of it’ (P8). Family caregivers generally had to help the patients communicate with the healthcare services and explain written information.

The patients experienced a lack of essential information about their own treatment. One patient who was seriously malnourished and almost blind could not obtain information about her nutritional treatment. During the first interview, she showed us an envelope she had received at the hospital; it contained a detailed nutritional plan that she was supposed to follow. She had no previous knowledge of this plan and could not remember if anyone at the hospital had informed her about it. She was not able to read it herself because of her poor eyesight. Her daughter, who did all her food shopping, had not been informed. She said, ‘They have made her a nutritional plan. It must be this one. They didn’t talk her to about it they just put it in the envelope with the rest of the papers’ (FC13). The patient was supposed to inform the home care services about this nutritional plan herself, but as she did not know she had received it, the care services were not informed and did not provide her with this necessary treatment.

The patients said that no one had talked to them about the risks associated with weight loss. ‘Interviewer: did the doctor tell you why it is important not to lose any more weight? Patient: No, he did not. He is so thin, and probably thinks I shouldn’t be any bigger’ (P8). Several of the patients were happy to finally have lost some weight: ‘I don’t understand why this has happened, but I am happy that my waistline is reduced. It doesn’t bother me that I lose some weight’ (P3).

The lack of mutual comprehension undermined the patients’ involvement in their healthcare decisions. Many experienced a lack of shared decision-making, and for some, this caused distress. For instance, decisions about their nutritional care were made without consulting them; receiving help against their will caused resistance: ‘Patient: They have started to prepare sandwiches for me, but I have told them to stop doing that. I can manage on my own. Interviewer: Did they start doing this after you came home from hospital? Patient: Yes, but I don’t know who have told them to do that’ (P9). Another patient said, ‘I don’t know what’s wrong with me. I just got a prescription for some new vitamin tablets. They cost a lot and I have refused to pay for them. I need an explanation of what’s going on’ (P3).

#### What about my opinion?

The patients experienced a way of communicating that lacked respect and sensitivity to their needs and values. The dialogue about food and meals with the home care services generally consisted of ‘What have you eaten?’, ‘What would you like to eat?’ and ‘It is important that you eat’. Some of the patients found this stressful. One family caregiver said, ‘They constantly urge her to eat and she says that she is fed up by it. That’s what they do, the people that visit her in the morning and the afternoon’ (FC14). Some felt like they were disrespected in the dialogue about food and meals or that food and medication was served to them without them agreeing to be helped or in a way that felt violating. One patient said, ‘Would you believe it, they went over and felt the casserole to see if it was warm. It was just like they didn’t believe us’ (P10). Another patient said, ‘They gave me some vitamin B and said you should take these. I don’t understand why. They just give me the pills and say take these, and then they leave’ (P2). Another patient experienced poor communication with the home care services when they just stopped visiting him without telling him why. He suspected that it was because they meant he could manage without help. The poor communication negatively affected the participants perceptions of the nutritional care.

### The role of family caregivers

#### Dependent on informal caregiver support to cover needs

Not being provided with care according to their individual needs at home resulted in patients being dependent on additional support from family caregivers, friends or neighbours. All of the family caregivers that participated in this study regularly did the grocery shopping for the patients. Occasionally, they brought homemade food and accompanied the patients during meals. They provided support that was essential for the patients’ nutritional well-being; still, they were generally not involved or informed by the healthcare services about nutritional care, and some lacked knowledge about the patients’ nutritional situations and needs: ‘I really think you need to be good at asking the right questions to get the information you need as a family caregiver. I myself may also seem a bit ignorant about these things?’ (FC1). One family caregiver described conflicting opinions between her and her mother about how much help she needed with meals at home: ‘They used to come four times a day, and then she told them that she only needed them to come three times a day. Sometimes when I come to visit around six o’clock she says, “I’m so hungry, they haven’t been here”. It was her that wanted them to come three times a day. But now I have asked them to change back to four visits a day so they can cook for her’ (FC6).

#### Lack of support to the family caregivers

Although the caregivers were generally thankful for the care that was provided to their loved ones, some family caregivers experienced disempowerment during meetings with the care services and felt they were not given the necessary support as caregivers. They felt it was demanding and sometimes exhausting caring for their loved ones because the care services were not able to provide care according to the patients’ needs. One daughter said, ‘I am her only family and I just can’t take it anymore. It is so exhausting, especially since she is so depressed’ (FC6). When the patients were living together with or close to family caregivers, home care services relied on the caregivers to provide nutritional care, which in some cases resulted in inadequate patient follow-up and burdened the family caregiver: ‘I experience that they keep on asking me to do things. And then I find out that that no, that is not my responsibility’ (FC6).

## Discussion

Our findings illustrate that the patients’ challenges with food and meals are related to aging and multi-morbidity as well as social setting and organisation of care. The patients describe nutritional care that lacks individualised considerations and an interaction with the care services that evokes feelings of disrespect and disempowerment. They also describe how loneliness negatively affects their meals. The nutritional care both in hospital and in home care seems to lack a person-centred approach that recognises and values the whole person, considers the needs and wishes of the patient and his or her family caregiver and accounts for the biological, social and psychological aspects that may affect a patient’s nutritional situation. Similar to our findings, Hope, Ferguson, Reidlinger and Agarwal [32] also found a mismatch between older patients’ nutritional needs and inflexible care systems and that the patients’ appetites and food intake were negatively affected by poor health, immobility and depression in addition to organisational aspects of care such as meal timings and not getting food according to their preferences.

The lack of mutual comprehension and shared-decision making undermined the patients’ involvement in their nutritional care. According to best practice guidelines for nutritional care, respecting the older person’s will and preferences should be of highest priority in nutritional care [3]. Studies do however, report that older patients commonly feel excluded from decision making, and that healthcare professionals rarely ask them about their preferences for care [16]. In contrast, one study found that older patients seldom explicitly expressed their emotional and existential needs, making it challenging for healthcare staff to meet these needs [33]. Algilani and colleagues [34] published a review of the literature on what constitutes participation from the perspective of older persons in the healthcare encounters. The older adults in their study, reported that in order to feel participation, healthcare professionals need to initiate and invite the older person into a relationship, on equal terms where participation is created. This involves a two-way communication, giving the older person enough time and taking responsibility to make the older adults feel that they are in the right place.

The patients in our study struggled between maintaining power, autonomy and privacy and being help-seeking individuals who had to let down their boundaries to receive help. Thus, it is important that the care receivers and the professionals agree on and negotiate the caring situation and how they both should engage in it. Marcus-Varwijk et al. [35] found that it was important for older adults to get to know their healthcare professionals and to build a relationship of trust to be able to discuss lifestyle issues such as nutritional needs and food habits. However, studies commonly report that healthcare providers find that the organisation and structure of care, such as lack of time and resources, lack of nutritional knowledge and skill and lack of communication between services, limit their ability to provide individualised nutritional care to older people in care settings [13, 36–39].

The participants in our study also felt that their healthcare providers communicated in a way that lacked respect and sensitivity to their needs and values. This may lead to a violation of their dignity, especially if the patient is ‘talked down to’ or spoken to ‘like a child’ and when healthcare providers disregard patients’ knowledge, skills, perceptions, concerns, needs and feelings [40]. Experiences of inclusion (or not) in the community of older persons arise in the context of interactions with others. If healthcare organizations and staff are to recognize and cultivate patients’ capabilities to experience inclusion in the community, they must give them the status of people who matter. This involves interacting with them in ways that signal they are valued as persons [41, 42]. That being said, healthcare providers sometimes find it difficult to establish a mutual dialogue with older patients. Their fixed and task-oriented agenda and limited time for conversation conflicts with the patients’ needs, resulting in ambiguous encounters, misinterpretation of the patients’ needs and dissatisfaction with care [43].

The patients in our study reported a lack of information, lack of understanding or problems remembering oral and written information about nutrition. Johnsson, Wagman, Boman and Pennbrant [44] found that the information nurses most commonly provided to older patients was related to their routine work, such as disease symptoms, test responses and drug changes. The authors emphasised the importance of the communication content and the value of asking didactic questions to improve the patients’ and relatives’ understanding of the information and to increase patient safety. Proper use of communication skills aims to increase healthcare providers’ understanding of patients’ individual needs, values and perspectives in order to give them the information they need to participate in their care and to build trust and understanding between healthcare providers and patients [45, 46]. Improving patients’ knowledge and awareness requires more than just providing information. In order find motivation and desire to cope with nutritional challenges, it is important that nutritional problems and their disadvantages are explained to patients, and there is a need for practical guidance on how to incorporate nutritional advice into daily life [45]. Thus, healthcare providers need to be effectively trained in communication skills in a systematic way, including practice dialogues with constructive feedback from instructors [45, 46]. This may lead patients to feel an increased sense of control over their own lives and contribute to their empowerment. Involving the older person in nutritional care may increase his or her desire to eat and enjoyment of food, thus decreasing the risk of weight loss, malnutrition and the other potential negative effects of poor food intake.

Patient and family engagement is emphasized as important in the delivery of nutritional care to older persons [47, 48]. However, the family caregivers in this study were not involved in care or were not informed about the patients’ nutritional needs. These findings are in line with other studies reporting that family caregivers often experience a lack of involvement and support when interacting with the healthcare system [18, 19]. Bélanger, Bourbonnais, Bernier and Benoit [49] found that on the one hand, nurses considered family caregivers as useful resources and partners in care, but on the other hand, considered them to be very demanding and time consuming. In order to maintain control over their time, they focused on the caring tasks at hand, and in some cases, limited their exchanges with family caregivers. Our results also show a discrepancy between patients and family caregivers’ perceptions of support needs. In a study by Holst et al. [50], patients reported that family caregivers were urging them to eat, and that they lacked understanding toward their situation. This could lead to decreased nutritional intake, underlining the patients’ need to be in control of their situations. Thus, if patients consent, family caregivers should be invited to join in on nutrition counselling and to participate in care planning in order to give the family caregiver increased insight into patients’ nutritional needs and provide them with knowledge on how to support and care for these needs. A systematic review by Marshall, Bauer, Capra and Isenring [51] found that involving informal caregivers in nutritional care could improve or prevent nutritional decline without increasing the care burden.

Some of the family caregivers expressed being exhausted from caring for their loved ones and felt that the care services relied on them to provide care that was not their responsibility. This is in accordance with a study showing that family members undertake caring tasks they are not trained or prepared for, and that patients and healthcare providers sometimes wrongly assume that family members are willing to take responsibility for care [52]. Simultaneously, family caregivers experience not being provided with information or the opportunity to be involved in care planning and inadequate education on the patient’s medical condition and additional care needs, such as special diets [52, 53]. The involvement and education of family caregivers seems to reduce the negative psychological impact on the family caregiver and to promote a smoother transition from hospital to home for the older patient [17, 52]. The feeling of being part of a team with the healthcare providers rather than making up for the lack of services may lessen the strain on family caregivers [54].

### Strengths and limitations

In qualitative research, the goal is to enhance the understanding of the phenomenon being studied [55]. This study gives a voice to older patients and their relatives on what is important to them when it comes to food, meals and nutritional care for older people. However, the study findings are limited by the specific demographics of the small sample size and the geographical location. Hence, the findings in this study cannot be generalised, but they may be transferred to similar situations or people [55].

The authors of this study have backgrounds within nutrition, nursing and physiotherapy. Throughout the study, we were conscious that our pre-understanding and backgrounds would influence the research process [26]. We tried to interpret the data as openly as possible and strove to provide a transparent description of the path from the data to the results. The involvement of multiple researchers with different backgrounds may also strengthen the design of a study, as they can supplement and contest each other’s statements [56].

Another strength is that interpretive description is a methodology developed to account for the clinical context of research in applied health disciplines with the aim to generate knowledge that is relevant. Thus, expert clinical knowledge is seen as solid grounding for this research design. On the other hand, a limitation may be that interpretive description is relatively new and less exhaustively discussed in the literature than many other methodologies. Thus there may be some ambiguity about how to conduct research using this method.

The patients’ vulnerable situations and poor health are further limitations to this study. Several of the patients were not feeling well and found it hard to talk; this might have influenced the quality of their interviews. The interviewer did her best to put the participants at ease and to listen empathetically and carefully. Several of them felt better at the second interview. By conducting two interviews, we were able to grasp the situation and experiences throughout a 30-day period, and this may be considered a strength of the study. However, the fact that some of the patients and family caregivers wanted to be interviewed together may have influenced the results by constraining them from freely describing their experiences of certain topics.

## Conclusions

This study demonstrates that nutritional problems among older patients are related to aging and multi-morbidity as well as social setting and organisation of care. The organisation of nutritional care and food provision to older people, depending on care, lack consideration for the individual’s values, needs and preferences. Older patients’ and their family caregivers’ needs and preferences should guide how nutritional care is provided.

## Relevance to clinical practice

New approaches are needed in nutritional care. Rather that applying society’s ‘one-size-fits-all’ solution to nutritional problems among older persons, the organisation of care needs to change to enable a more person-centred approach. Well-managed nutritional care should systematically map patients’ nutritional needs and preferences as well as pay attention to the cultural and social meaning that food and meals have in people’s lives. Care should be flexible and have the capacity to change according to individual variation and changing needs. Attention should also be placed on educating healthcare professionals about how to communicate with older patients and their caregivers in an effective and respectful manner. Expanding the time, availability and engagement of allied healthcare providers may promote better communication and care. The predictability of personnel and the timing of care are also important factors that need urgent attention.

## Data Availability

Not applicable.

## Abbreviation

MNA: Mini Nutritional Assessment
